# Small RNA Sequencing in Cells and Exosomes Identifies eQTLs and 14q32 as a Region of Active Export

**DOI:** 10.1534/g3.116.036137

**Published:** 2016-10-31

**Authors:** Emily K. Tsang, Nathan S. Abell, Xin Li, Vanessa Anaya, Konrad J. Karczewski, David A. Knowles, Raymond G. Sierra, Kevin S. Smith, Stephen B. Montgomery

**Affiliations:** * Biomedical Informatics Program, Stanford University, California 94305; † Department of Pathology, Stanford University, California 94305; ‡ Department of Genetics, Stanford University, California 94305; § Department of Radiology, Stanford University, California 94305; ** Stanford PULSE Institute, SLAC National Accelerator Lab, Menlo Park, California 94025

**Keywords:** extracellular vesicles, miRNA, piRNA, gene regulation

## Abstract

Exosomes are small extracellular vesicles that carry heterogeneous cargo, including RNA, between cells. Increasing evidence suggests that exosomes are important mediators of intercellular communication and biomarkers of disease. Despite this, the variability of exosomal RNA between individuals has not been well quantified. To assess this variability, we sequenced the small RNA of cells and exosomes from a 17-member family. Across individuals, we show that selective export of miRNAs occurs not only at the level of specific transcripts, but that a cluster of 74 mature miRNAs on chromosome 14q32 is massively exported in exosomes while mostly absent from cells. We also observe more interindividual variability between exosomal samples than between cellular ones and identify four miRNA expression quantitative trait loci shared between cells and exosomes. Our findings indicate that genomically colocated miRNAs can be exported together and highlight the variability in exosomal miRNA levels between individuals as relevant for exosome use as diagnostics.

Most cell types, including those grown in culture, produce and release exosomes (Raposo and Stoorvogel 2013), and compelling evidence that exosomes mediate intercellular communication exists in multiple contexts including immune modulation (Zitvogel *et al.* 1998; Kim *et al.* 2005; Segura 2005; Admyre *et al.* 2007; Alexander *et al.* 2015), cancer proliferation (Meckes *et al.* 2010; Hood *et al.* 2011; Peinado *et al.* 2012; Tadokoro *et al.* 2013; Boelens *et al.* 2014; Costa-Silva *et al.* 2015; Fong *et al.* 2015), and neuronal activity (Frühbeis *et al.* 2013; Chivet *et al.* 2014). These extracellular vesicles range from 30 to 100 nm in diameter and are secreted by exocytosis into biological fluids and culture medium when an endosome harboring multiple vesicles fuses with the plasma membrane (Harding *et al.* 1983; Pan *et al.* 1985). They enable communication by shuttling heterogeneous cargo, including DNA, RNA, proteins, and lipids, from their cell of origin to targeted recipients (Harding *et al.* 1983; Valadi *et al.* 2007; Mittelbrunn *et al.* 2011; Thakur *et al.* 2014). For instance, *in vitro* studies have revealed that exosomal miRNAs can repress known target genes in recipient cells (Hergenreider *et al.* 2012; Montecalvo *et al.* 2012; Umezu *et al.* 2013; van Balkom *et al.* 2013).

Studies in diverse cell types have demonstrated that the RNA content of exosomes differs from that of their parent cells (Nolte-’t Hoen *et al.* 2012; Villarroya-Beltri *et al.* 2013; Koppers-Lalic *et al.* 2014; Squadrito *et al.* 2014). However, these studies compared small numbers of samples, which limited their power to detect differentially expressed genes. Modest sample sizes have also precluded the investigation of interindividual variability in exosome cargo, which may include genetically driven differences. We can identify such individual-specific differences by identifying genetic variants associated with gene expression, known as expression quantitative trait loci (eQTLs). Most protein-coding genes, as well as some miRNAs, have at least one known eQTL in cells (Borel *et al.* 2011; Lappalainen *et al.* 2013; Battle *et al.* 2014; Huan *et al.* 2015). However, it remains unknown whether exosomes mirror cellular expression differences between individuals, and understanding interindividual variability is crucial as the field moves toward using exosomes in diagnostics.

Most eQTL studies rely on cohorts of ≥60 unrelated individuals to detect significant effects, but we have previously shown that we can leverage large nuclear families to identify eQTLs from smaller samples (Li *et al.* 2014). Here, we sequenced the small RNA from the lymphoblastoid cell lines (LCLs) and associated exosomes of a 17-member family spanning three generations. This represents, to our knowledge, the largest set of paired cell and exosome samples analyzed to date. We aimed to comprehensively quantify differences in small RNA between cells and exosomes, to assess interindividual variability, and to establish whether genetic variants influence exosome content. While it has previously been shown that miRNAs are differentially present in cells and exosomes, we discovered that exosomes can export entire genomic clusters of miRNAs. Furthermore, by using publicly available whole genome sequences, we performed the first eQTL study in exosomes to elucidate the impact of genetic variation on the small RNA in exosomes.

## Materials and Methods

### Samples and genotype data

CEPH/UTAH family EBV-transformed peripheral blood B LCLs (catalog no. XC01463) were purchased from the Coriell Institute for Medical Research. The samples are from a complete three-generation pedigree that includes four grandparents, two parents, and 11 children. Variant calls from whole genome sequencing data for the 17 individuals were obtained from Complete Genomics (Analysis Pipeline v.2.0.0).

### Cell culture and exosome isolation

**Figure 1:**
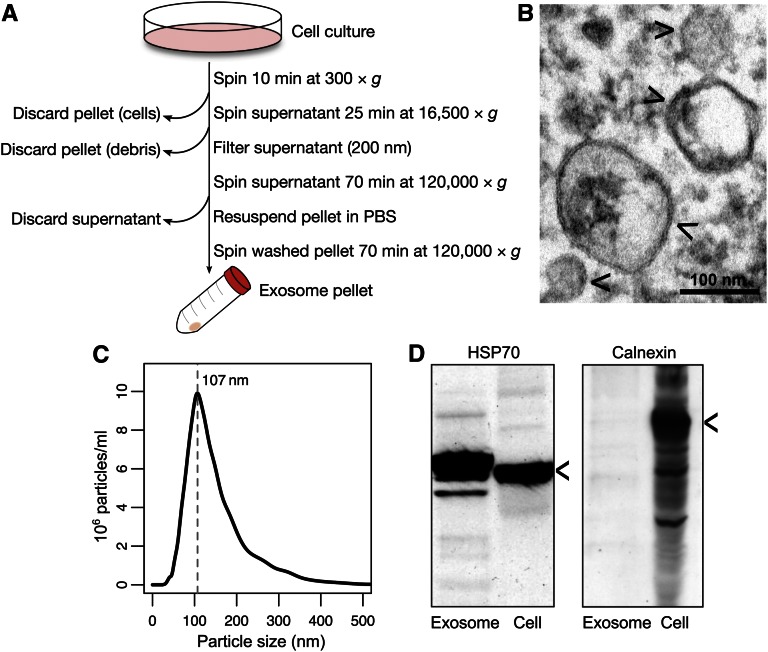
LCL exosome isolation procedure yields vesicles characteristic of exosomes. (A) Flow diagram of the exosome isolation procedure. All centrifugations were performed at 4°. (B) Transmission electron microscopy of isolated LCL exosomes. Four arrowheads denote isolated examples. Bar, 100 nm. (C) Example NanoSight tracing of LCL exosomes from a representative sample. For that sample, the maximal concentration of exosomes was at 107 nm diameter, as indicated by the dashed line. (D) Western blots of 50 µg total protein lysates from LCLs or their isolated exosomes hybridized with HSP70-specific (left) or calnexin-specific (right) antibodies. Arrowheads indicate expected bands at 70 and 90 kDa, respectively.

LCLs were grown in RPMI 1640 supplemented with 10% fetal calf serum (Gibco, Life Technologies) and 1× penicillin/streptomycin (Life Technologies) in humidified 5% CO_2_. Cell cultures were initiated at densities of 500–750 × 10^5^ cells/ml and allowed to grow to 150 × 10^6^ cells, achieving a maximum density of 2 × 10^6^ cells/ml at collection. Fetal calf serum was depleted of bovine exosomes before use by overnight centrifugation at 120,000 × *g*. The exosome isolation procedure is depicted in Figure 1A and described in detail here. LCLs were pelleted at 300 × *g* for 10 min and saved for cell miRNA isolation. The remaining growth medium was centrifuged at 16,500 × *g* for 25 min and then filtered through a 200 nm Acrodisc (PAL Corp., Ann Arbor, MI). The filtered supernatant was centrifuged at 120,000 × *g* for 70 min to pellet exosomes (Ti45 rotor). Pelleted exosomes were washed 1× in cold PBS and centrifuged again at 120,000 × *g* for 70 min (TLA 100.3 rotor). All centrifugations were performed at 4°.

### Exosome characterization

Exosomes isolated from LCLs were characterized by Western blot, electron microscopy, and NanoSight nanoparticle tracking analysis (Malvern, Worcestershire, UK). For Western blotting, ∼50 µg total cell and exosome proteins were denatured with 1× sample buffer, reduced with β-mercaptoethanol, and boiled for 5 min. Proteins were resolved on 10% PAGE gels, blotted to nitrocellulose, and probed with antibodies recognizing HSP70 and calnexin. The Stanford University Cell Sciences Imaging Facility performed transmission electron microscopy of isolated intact exosomes. Nanoparticle tracking analysis software (update 2.3) was used to determine the average size of isolated exosomes using a NanoSight LM10-HS at the Stanford PULSE Institute.

### Small RNA isolation and cDNA library construction

Small RNA from TRIzol-lysed LCLs and exosomes was isolated from 1 µg total RNA and analyzed for integrity using a Bioanalyzer 2100 Total RNA 6000 Nano Kit. cDNA libraries were produced using the TruSeq Small RNA Sample Prep Kit (Illumina, San Diego, CA) per manufacturer’s protocol, with the following modifications: (1) final PCR amplifications were 16 cycles for exosome cDNA and 14 cycles for cell cDNA, (2) instead of gel purification using PAGE, PCR-amplified small RNA libraries were gel-purified with 3% NuSieve GTG low-melt agarose (Lonza, Rockland, NY). Target bands of 147–157 nt, containing an adapter sequence of 125 nt, were excised and column purified (Qiagen, Germantown, MD). All cDNA libraries were indexed with Illumina adapters.

### Transcriptome sequencing

Small RNA was sequenced on two lanes (one with cell samples and the other with exosome samples) of a single Illumina HiSeq 2000 flow cell as 36 bp single-end reads. This sequencing setup was chosen to maximize our power for eQTL discovery but meant that lane effects were confounded with the effect of interest for differential expression. Therefore, we resequenced the libraries to lower depth on two runs of an Illumina MiSeq, where each run had both the cells and exosome samples for half the individuals.

### Quantification and cluster generation

Transcript quantification steps are depicted in Supplemental Material, Figure S1 and described here. Sequencing adapters were trimmed with Trimmomatic (v0.27) and were also provided to Novoalign (v3.00.02, www.novocraft.com), which handled incomplete adapter trimming while mapping. The reads were mapped in two steps where they were first mapped to the mature sequences in miRBase (release 20) (Kozomara and Griffiths-Jones 2014) and piRNABank (Sai Lakshmi and Agrawal 2007), then unmapped reads were aligned to the human reference genome (hg19). Novoalign was used for both mapping steps, with the parameters -l 18 -s 1 -a <adapter> -R 0 -r Random -t 0. The reads that mapped to the reference genome were assigned to transcripts annotated in Gencode (version 17), miRBase, or piRNABank using HTSeq (v0.5.4p3) (Anders *et al.* 2014). The read counts from the first mapping step were added to those generated by HTSeq to get a single read count per transcript.

An miRNA cluster is defined as a set of miRNAs where every cluster member is within 10 kb of at least one other member. We identified all such clusters in the human genome using miRBase annotations. For each cluster, we summed the read counts of all mature miRNAs mapping to that cluster, excluding mature miRNAs that could have originated from multiple clusters. We used the resulting matrix for cluster-level differential expression. Notably, since many miRNAs do not cluster with any others, their mature and cluster counts are identical. As such, mature and cluster MA plots are visually similar, though not identical.

### Differential expression analysis

For each sample, we computed the proportion of reads mapping to each class of RNA. We compared the small RNA composition of cells and exosomes by averaging the proportions of the 17 individuals for each compartment. We tested for a difference between the compartments with a paired two-sided *t*-test and corrected for multiple testing using the Bonferroni method. When comparing the quantities of different small RNA types, we considered miRNAs that were annotated in either Gencode or miRBase, but for all other analyses we only used the higher confidence set of miRNAs annotated in miRBase, which accounts for >99.5% of the reads mapped to miRNAs. Transcript- and cluster-level differential expression between cells and exosomes was performed separately for miRNA, miRNA clusters, and piRNA using DESeq2 (v1.6.3) (Love *et al.* 2014), which uses a negative-binomial distribution to directly model counts obtained from RNA sequencing. We modeled the mean expression as a function of whether the sample came from cells or exosomes and controlled the false discovery rate (FDR) at 1% with the Benjamini–Hochberg adjusted *p*-values (Benjamini and Hochberg 1995) reported by DESeq2. To confirm that the differential expression between cells and exosomes was not due to lane effects, we compared the initial HiSeq2000 sequencing data to the resequencing data (see *Transcriptome sequencing*) both with and without the sequencing run explicitly included in the DESeq2 model. Because the resequencing data had lower sequencing depth and therefore lower power to detect differential expression, we also compared with downsampling the initial data to 10%, which matched the depth of the resequencing data.

### Replication of chromosome 14q32 miRNA cluster differential expression

We processed the raw reads counts from an exosome sequencing study in HeLa cells (Honegger *et al.* 2015) following the same procedure we used on our data to identify differentially expressed miRNA. We also downloaded the differentially expressed results produced by EdgeR from another sequencing study in B cell lines (Koppers-Lalic *et al.* 2014). Since the replication data sets used miRBase releases 18 and 19 whereas we used release 20, we confirmed that the annotations of the 14q32 cluster hairpins did not change between releases 18 and 20.

### Motif enrichment

For cells and exosomes separately, we identified mature miRNA sequences that were significantly enriched in that compartment (differential expression FDR < 1%). Then, for the separate exosome-enriched and cell-enriched sets, we defined all other annotated miRNAs as background (exosome-enriched *vs.* all nonexosome-enriched and cell-enriched *vs.* all noncell-enriched, separately). To identify cell- or exosome-enriched miRNA motifs, we applied the MEME and DREME algorithms from the MEME Suite toolset (v4.11.1) (Bailey *et al.* 2009) with default parameters, using windows of 9–13 and 3–8 bp motifs, respectively.

### Nontemplated nucleotide addition analysis

To profile nontemplated nucleotide additions (NTAs) from sequencing data, we remapped reads to miRBase (release 20) hairpins using Bowtie2 (v2.2.4) (Langmead and Salzberg 2012) in local alignment mode with sensitive parameters (-D 50 -R 5 -N 0 -L 10 -i C,1,0--score-min = C,32,0), requiring a minimum alignment of 16 bp, without penalizing soft clipping. We extracted 5′- and 3′-NTAs separately from the resulting BAM files using the CIGAR string. For every sample, we counted the number of occurrences of each NTA (*e.g.*, “-AG”) for each miRNA hairpin (*e.g.*, “hsa-miR-21”), extracted counts for each 1- or 2- bp NTA, and computed the percentage of all miRBase-aligned reads that possessed each NTA. To further analyze a given NTA or set of NTAs, for each sample we computed the percentage of all reads with that NTA originating from each miRNA hairpin.

### eQTL mapping

From the variants identified in the Complete Genomics whole genome sequencing data, we removed any variants with Mendelian inconsistencies and used the haplotype blocks we had previously inferred for the 11 children (Li *et al.* 2014). Briefly, the haplotype blocks were defined as segments between any two recombination points in the children. For each child, we then determined which of the two possible maternal and two possible paternal haplotypes that child received.

To map miRNA and piRNA *cis*-eQTLs, we used the linear model described in Li *et al.* (2014) to test for an association between a transcript’s expression level in the children and the parental haplotypes they inherited at the transcript’s locus:

$$\log\left(Y_{i}+\text{1}\right)∼\mu +\beta_{p}\;p_{i}+\beta_{m}\;m_{i}$$

where *Y_i_* is the DESeq2-normalized expression in child *i*, *µ* is the intercept, and *p_i_* and *m_i_* are the paternal and maternal haplotypes (encoded as 0 or 1) of the child at the locus containing the transcript being tested. The coefficients *β_p_* and *β_m_* represent the effect sizes of the parental haplotypes. Each association was tested separately in cells and exosomes and the model fit (*R^2^*) was used to obtain *p*-values for the associations. Only transcripts with a median normalized expression of at least 10 in the given compartment were tested. Transcripts mapping to multiple loci, as well as ones containing variants (which could bias mapping given the short read length), were also excluded.

The Pearson correlation of the effect sizes in cells and exosomes was used to assess the overall sharing of genetic effects between cells and exosomes. To identify specific instances of shared miRNA eQTLs, we intersected the cell and exosome miRNA eQTLs discovered at a 20% FDR.

### Accounting for nongenetic factors in miRNA eQTL discovery

Several factors were assessed to rule out likely nongenetic explanations for the shared miRNA eQTLs to increase our confidence that we were detecting true genetic effects. We confirmed that the haplotypes of the candidate-shared miRNA eQTLs were not confounded with sex and were not explained by mapping differences. Spurious eQTLs driven by different mapping rates for the two haplotypes would be detectable by differential quantities of k-mers in the unmapped reads. Therefore, for each sample, 10-mers in the unmapped reads were counted with Jellyfish (v2.1.4) (Marçais and Kingsford 2011) and normalized by the number of sequenced reads from that sample. We verified that none of the candidate-shared eQTLs had 10-mer counts that differed significantly between the haplotypes in the opposing direction of the eQTL effect (one-sided *p*-value ≤ 0.05, Wilcoxon rank sum test). Finally, we checked whether the haplotypes of shared miRNA eQTLs were associated with many miRNAs genome-wide in both cells and exosomes. An excess of associations may indicate the haplotype is correlated with a technical factor influencing the expression of many miRNAs. We ranked haplotypes by their number of significantly associated transcripts (nominal *p*-value ≤ 0.05) to assess which haplotypes are most likely to correlate with a technical factor. In our data, we noticed that the haplotype containing the hsa-miR-151a products is ranked 13th among 697 haplotypes in terms of the number of miRNAs genome-wide that it is associated with in cells (at a nominal significance level of 0.05). It is possible that this haplotype’s segregation pattern among the children is correlated with a technical factor influencing the expression levels of many miRNAs, but we think this is unlikely since the eQTL replicates both in exosomes and in a different cohort.

### Data availability

RNA sequencing fastqs and raw read counts are available at GEO with the accession number GSE74759. File S1 includes the legends for Table S1, Table S2, and Table S3, which include read mapping statistics, differential expression results, and eQTL data and results, respectively.

## Results

### Isolation and characterization of exosomes in LCLs

We isolated exosomes following the procedure outlined in Figure 1A. To validate this protocol, we subsequently assayed the size and protein content of extracted exosomes. We first imaged exosome pellets by transmission electron microscopy and confirmed that we isolated vesicles with bilayered membranes of varying sizes, averaging roughly 100 nm (Figure 1B). We next applied nanoparticle tracking to confirm the vesicle sizes assessed by microscopy and found that most vesicles had a diameter just above 100 nm (Figure 1C). Finally, we made total protein lysates from both exosomes and parent cells and probed for enriched proteins by Western blotting. We detected HSP70 in lysate fractions of both cells and exosomes, but only resolved the endoplasmic reticulum-specific protein calnexin in the cell fraction (Figure 1D), which agreed with previous biochemical characterizations of exosomes (Baietti *et al.* 2012).

### Cells and exosomes differ in small RNA composition

**Figure 2:**
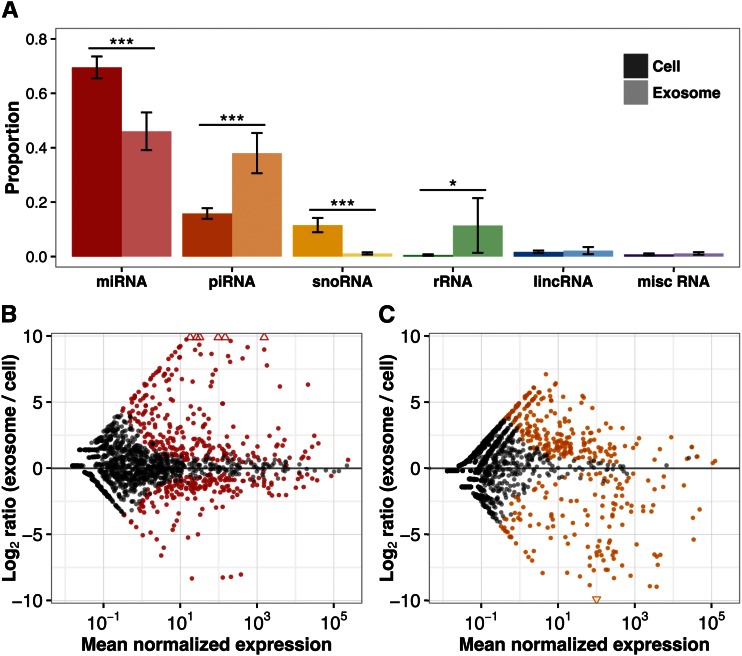
Cells and exosomes differ in their small RNA profiles. (A) Small RNA composition of cells and exosomes averaged over the 17 individuals. Error bars show the SD. Paired two-sided *t*-tests were used to compare the cell and exosome proportions for each miRNA type and the *p*-values for the six tests were corrected by the Bonferroni method. Asterisks denote the significance of the corrected *p*-values: *** *p* < 1 × 10^−7^; * *p* = 0.002. (B and C) MA plots for miRNA (B) and piRNA (C) where each point represents one transcript. Above the center horizontal line are transcripts that were relatively more abundant in exosomes and below are the ones that were present in relatively higher quantities in cells. The transcripts that were significantly differentially expressed at a 1% FDR are colored. Triangles represent points that fall outside the plotted area. lincRNA, long intergenic noncoding RNA; miRNA, micro RNA; misc. RNA, miscellaneous other RNA; piRNA, Piwi-interacting RNA; rRNA, ribosomal RNA; snoRNA, small nucleolar RNA.

We sequenced small RNA libraries for cells and exosomes of the 17 family members to an average depth of 5.9 million reads after removing reads that were too short after adapter trimming (Table S1). By comparing cells with exosomes, we noticed broad differences in the distribution of RNA biotypes. Cells expressed proportionally more miRNA and small nucleolar RNA, while exosomes contained a greater proportion of Piwi-interacting RNA (piRNA) and ribosomal RNA (Figure 2A). The proportion of other RNAs was similar between cells and exosomes. While we observed differences between cells and exosomes for four RNA classes, we focused on miRNA and piRNA for the remainder of the analyses, since these two types of RNA are the most abundant and corresponded to the target lengths of our small RNA isolation step at ∼22 and 24–30 nucleotides long, respectively (Ha and Kim 2014).

### Abundant differential expression of miRNA and piRNA between cells and exosomes

We tested whether individual miRNAs and piRNAs were selectively exported in exosomes or retained in cells (Figure 2, B and C). Of the 1739 miRNAs expressed in at least one sample, cells and exosomes differentially expressed 433 (25%) at an FDR of 1%. Cells expressed 187 miRNAs at higher levels, while exosomes contained proportionally more of the remaining 246 miRNAs (Table S2A). Of the 3422 piRNAs expressed, we found 374 (11%) were differentially expressed, with 135 being more highly expressed in cells and 239 more frequent in exosomes compared with cells (Table S2B). Since the cells and exosomes were sequenced on different lanes, confounding the contrast of interest with potential lane effects, we resequenced the libraries this time batching cells and exosomes from the same individual together. Our resequencing data confirmed the differential expression results from our initial sequencing run, establishing that any lane effects were minimal (Figure S2). Our results highlight clear and widespread differences in the levels of particular miRNAs and piRNAs between cells and exosomes.

### Exosomes export a large miRNA cluster on chromosome 14q32

**Figure 3:**
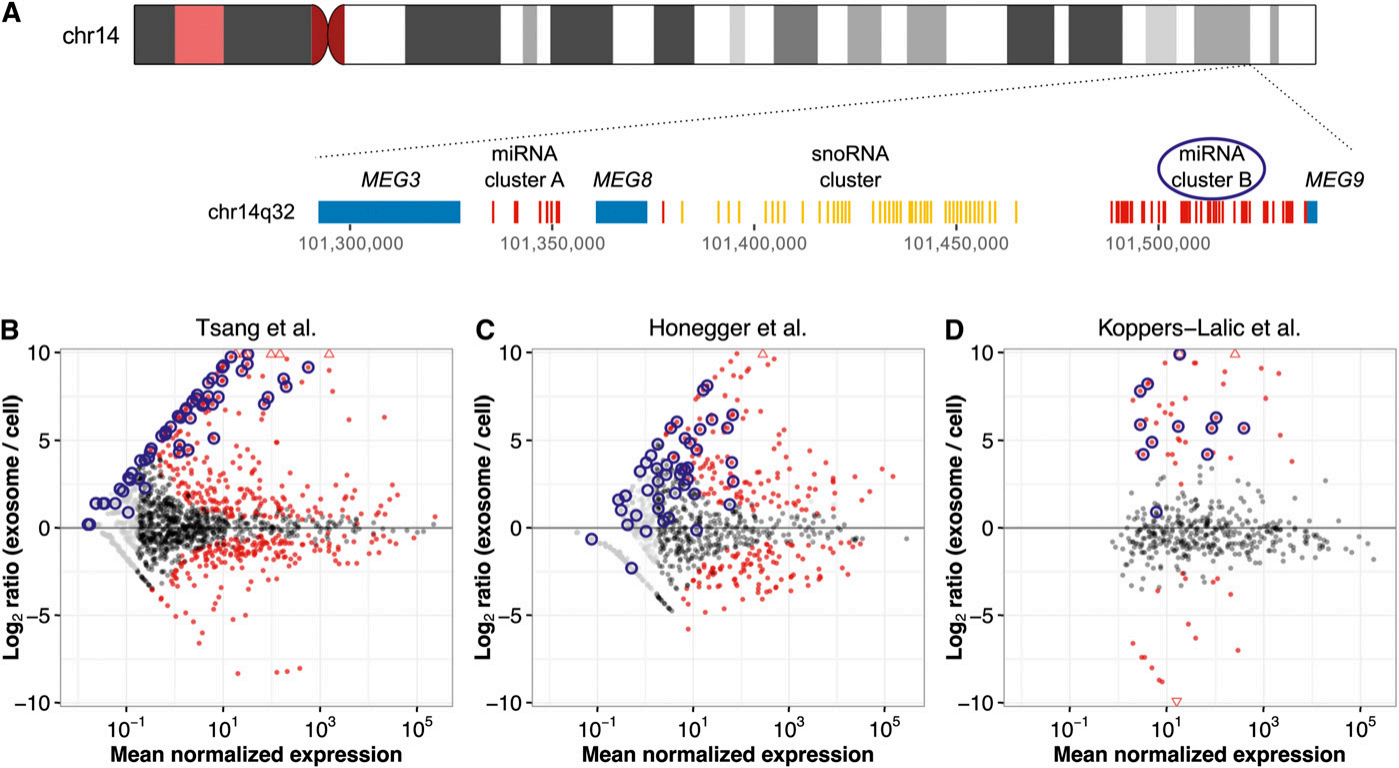
A large miRNA cluster on chromosome 14q32 is exported in exosomes. (A) Diagram of the 14q32 locus, which contains two miRNA clusters denoted as cluster A and cluster B that comprise 15 and 74 mature miRNAs, respectively. These miRNA clusters are flanked by lincRNAs and separated by a lincRNA and an snoRNA cluster. (B) MA plot of our miRNA differential expression results (*n* = 34) with miRNAs from the larger miRNA cluster on 14q32 circled in dark blue. DESeq2 uses independent filtering to reduce the number of explicit differential expression tests it runs (Love *et al.* 2014). miRNAs that were not differentially expressed are depicted in light gray if they were removed by independent filtering and in dark gray otherwise. Significantly differentially expressed miRNAs (FDR = 1%) are colored in red. (C and D) Replication of the overrepresentation in exosomes of miRNAs from the large cluster on 14q32 using HeLa cell data (*n* = 5) from Honegger *et al.* (2015) (C) and B cell line data (*n* = 6) from Koppers-Lalic *et al.* (2014) (D). Note that the Koppers-Lalic *et al.* data were tested for differential expression using EdgeR, so the *x*-axis is in counts per million (instead of in DESeq2 normalized counts). Since only miRNAs that were explicitly tested for differential expression were reported, no light gray points appear in D.

Many miRNAs are in close genomic proximity to other miRNAs, and are sometimes transcribed as long polycistrons with multiple hairpins (Altuvia *et al.* 2005). If clustered miRNAs have correlated counts, then analyzing cluster-level rather than mature miRNA counts may be a more powerful approach. Accordingly, we generated cluster-level counts for each sample and tested for differential cluster expression between cells and exosomes. While the cluster results broadly mimicked the mature miRNA results (as many miRNAs are in their own individual cluster), two large miRNA clusters appeared to be selectively exported in exosomes (Figure S3). Most of the 42-hairpin 14q32 miRNA cluster, which yields 74 mature miRNAs, was massively exported into exosomes while being nearly undetectable in cells (Figure 3, A and B). We additionally confirmed that miRNAs from this cluster were enriched in exosomes in data from two independent studies (Koppers-Lalic *et al.* 2014; Honegger *et al.* 2015) that sampled additional cell types, such as HeLa cells (Figure 3, C and D).

### Selective miRNA export in exosomes is incompletely explained by known miRNA sorting mechanisms

The mechanism controlling coordinated export of the 14q32 cluster in exosomes is unknown. In the normal miRNA biogenic pathway, primary miRNAs (pri-miRNAs), including polycistronic miRNAs, are transcribed then cleaved into individual hairpins before nuclear export (Bartel 2004). Therefore, even if the cluster is transcribed as a polycistronic pri-miRNA—a hypothesis put forward by Seitz *et al.* (2004) that remains to be rigorously verified in humans—the component hairpins would typically be physically separated before entering the cytoplasm. We explored two mechanisms derived from recent studies (Villarroya-Beltri *et al.* 2013; Koppers-Lalic *et al.* 2014) that could explain exosome miRNA sorting for this cluster in particular, or for all differentially expressed miRNAs: (1) that a short sequence motif directs exosome loading, and (2) that NTAs to the 3′ end of the mature miRNAs favor inclusion in exosomes or retention in cells.

We searched for exosome- or cell-specific motifs using the MEME algorithm, but did not identify previously characterized motifs (Villarroya-Beltri *et al.* 2013). The 14q32 cluster dominated the exosome signal so strongly that the only significant motif was a sequence shared by most miRNAs in that cluster, and the cell signal was similarly dominated by the sequence similarities within the let-7 and mir-17 miRNA families. We then profiled and analyzed 3′ NTAs in all samples. On average, 80% of miRNA reads in both compartments lacked NTAs. This was true for both the 14q32 cluster as well as for the set of all mature miRNAs. We failed to detect substantial changes in the frequencies of NTAs between compartments, except for poly-adenylation, which was significantly higher in cells than exosomes. However, the adenylation of a small number of highly abundant miRNAs, rather than consistent changes in adenylation frequencies across many miRNAs, appeared to drive that effect (Figure S4). Taken together, these data suggest that neither known sequence motifs nor 3′-NTAs explain 14q32-encoded miRNA export or our differential expression results more generally.

### Interindividual variability in miRNA and piRNA expression profiles

**Figure 4:**
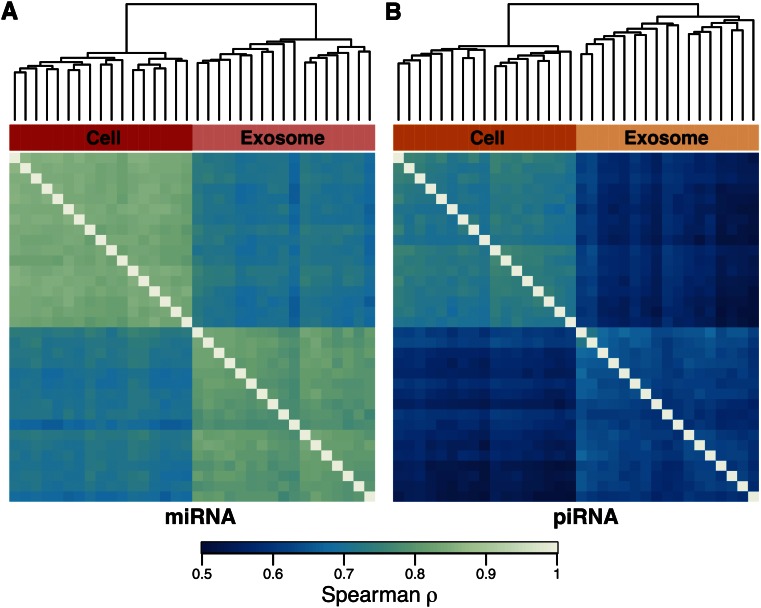
Cells and exosomes cluster by their miRNA and piRNA expression profiles. Hierarchical clustering of samples by the Spearman correlation coefficients of (A) miRNA and (B) piRNA expression. Samples cluster by compartment, confirming that cells and exosomes have distinct expression profiles. On average, the correlations between cell samples are higher than between exosome samples for both miRNA (0.83 *vs.* 0.80) and piRNA (0.72 *vs.* 0.61) (two-sided Wilcoxon rank sum test, *p* < 1 × 10^−15^ for miRNA and piRNA).

We clustered samples hierarchically, according to the Spearman correlation of their miRNA and piRNA expression, and found that samples from the same compartment cluster together based on both their miRNA and piRNA expression profiles (Figure 4 and Figure S5). Notably, there was significantly more variability between exosomes samples than between cells samples for both miRNA and piRNA, though the difference was small for miRNA, as well as more variability overall for piRNA compared with miRNA (Figure 4 and Figure S5).

### Cells and exosomes can share miRNA eQTL

Genetic variants associated with the expression of particular genes, known as eQTLs, have been studied in multiple cell types and tissues as a common source of gene expression variability between individuals (GTEx Consortium 2015). However, to our knowledge, it remains unknown whether genetic variation influences exosome content. To evaluate this, for each unique miRNA and piRNA, we tested for an association between the expression in the 11 children and the parental haplotypes they inherited, using a linear model (Table S3). The excess of small *p*-values for miRNA eQTL tests in cells suggests that genetic differences may explain a portion of the variability observed between individuals (Figure S6A). We observed the same but weaker signal in exosomes, but uncovered little evidence of piRNA eQTLs in either cells or exosomes (Figure S6, A and B).

**Figure 5:**
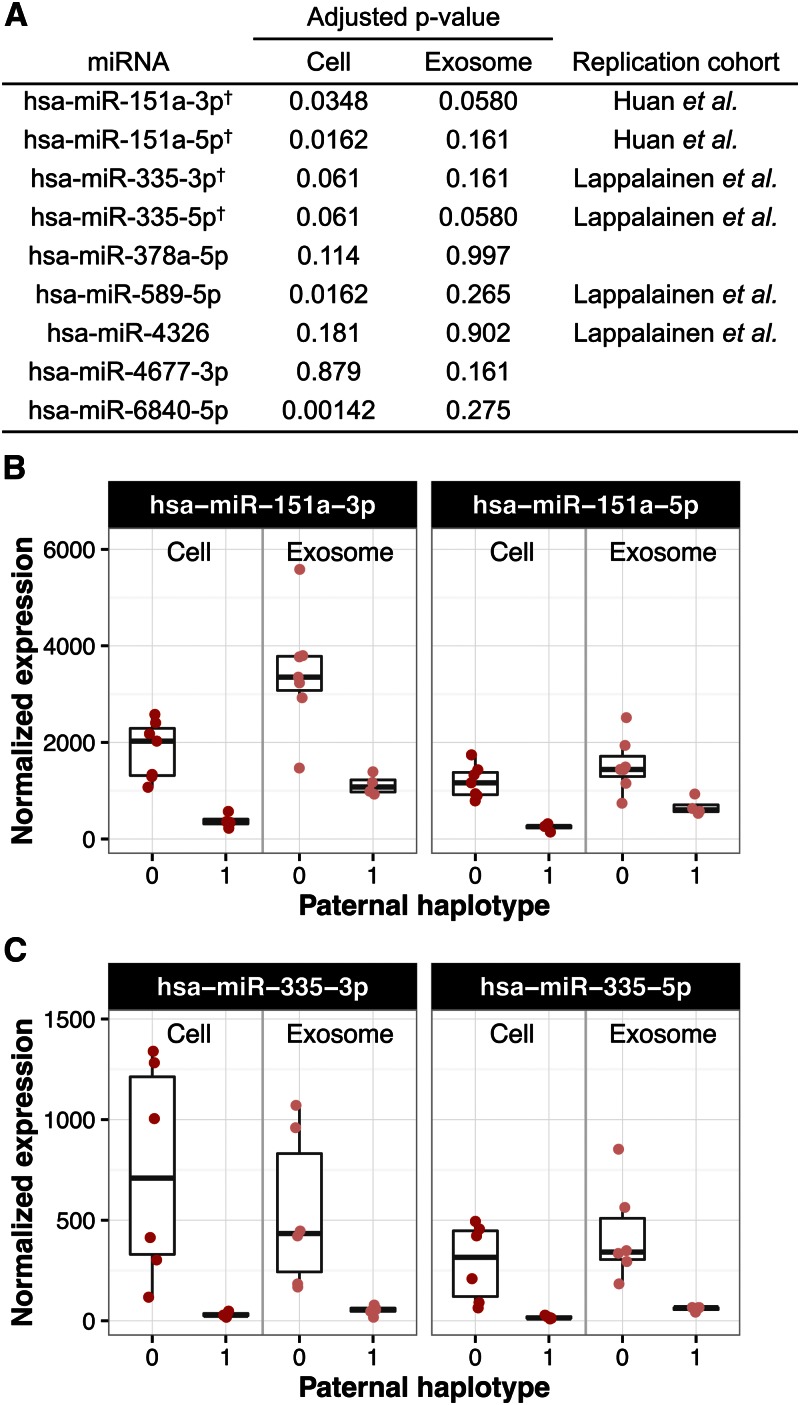
Shared miRNA eQTL between cells and exosomes. (A) Table of all miRNAs with an eQTL at FDR ≤ 20% in either cells or exosomes. The four miRNAs that pass the FDR threshold in both cells and exosomes are marked with a dagger and depicted in (B and C). (B and C) Putative shared miRNA eQTLs. The normalized expression levels of the 11 children are shown in cells and exosomes for both products of hsa-miR-151a (B) and hsa-miR-335 (C). The expression values are segregated by their inherited paternal haplotype, denoted as 0 or 1. The maternal haplotypes are not depicted because they did not show a strong association with expression.

We observed that the miRNA eQTL effect sizes of both parental haplotypes were significantly correlated between cells and exosomes, hinting at shared genetic effects on expression (Figure S7). To explore this further, we intersected the list of significant miRNA eQTLs from cells and exosomes at an FDR of 20% (Figure 5A). This identified four mature miRNAs, the pairs of products from hsa-miR-151a and hsa-miR-335, as putative shared miRNA eQTLs (Figure 5, B and C). The effect sizes are comparable within each pair of products, which is consistent with a *cis*-regulatory variant controlling the transcription of the miRNA precursors. Furthermore, the hsa-miR-151a and hsa-miR-335 products were previously identified as having an eQTL in cells by Huan *et al.* (2015) and Lappalainen *et al.* (2013), respectively.

## Discussion

We isolated extracellular vesicles from the LCLs of a large family, then verified they were exosomes by three orthogonal methods. By sequencing the small RNA from both cells and exosomes, we not only confirmed previous observations that exosomes selectively export specific transcripts from the cells that release them (Nolte-’t Hoen *et al.* 2012; Villarroya-Beltri *et al.* 2013; Koppers-Lalic *et al.* 2014; Squadrito *et al.* 2014), but also discovered that a large miRNA cluster on chromosome 14q32 is exosome-specific. We observed that exosome expression profiles tend to be more variable between individuals than cellular ones. Finally, we provide the first evidence that exosomes reflect a subset of genetically driven cellular miRNA expression differences.

Our large sample for differential expression analysis provided ample power to detect differences between cells and exosomes, and our results support the hypothesis of directed RNA export in exosomes. Indeed, we report almost an order of magnitude more differentially expressed transcripts than a previous exosome sequencing study in LCLs (Koppers-Lalic *et al.* 2014). Overall, the relative proportions of the RNA biotypes we measured in cells and exosomes agree with previous work in immune cell types (Nolte-’t Hoen *et al.* 2012; Koppers-Lalic *et al.* 2014) when we consider the types of RNA in common between the studies. One exception is that Koppers-Lalic *et al.* (2014) observed the same relative quantity of piRNA between cells and exosomes, while we observed proportionally more piRNA in exosomes. This discrepancy is probably explained by differences in our mapping strategies. Several piRNA reside in multiple genomic loci and therefore can be challenging to map uniquely to the reference genome. Since we specifically aligned reads to piRNABank as well as to the reference genome, our method can capture more piRNA reads, allowing us to perform a more sensitive analysis of this type of RNA.

We also discovered that a large cluster of miRNAs on 14q32 is preferentially present in exosomes while being virtually undetectable in cells. This constitutes the first report of a large set of miRNAs with a shared genomic locus almost completely exported by exosomes. The maternally expressed 14q32 locus contains two clusters of eight and 42 miRNA hairpins, spanning 200 kb. Multiple studies have linked expression changes in these miRNAs to human diseases and cancers (Sarver *et al.* 2013; Kameswaran *et al.* 2014; Manodoro *et al.* 2014), and have described the effect of deleting larger regions around the 14q32 locus, including 14q uniparental disomy (Temple *et al.* 1991; Ogata and Kagami 2016). Many of these studies measured and associated cellular miRNA levels with a range of molecular and cellular phenotypes, such as epigenetic modification and disease progression. Our results suggest that cellular miRNA levels may inaccurately reflect 14q32 locus expression if exosomes export virtually all resulting miRNA products; indeed, in our data, the locus would appear silenced if evaluated solely by our cellular miRNA samples. While we observed replication of this cluster-level differential expression in HeLa cells, it remains important for future studies to replicate our observations in primary cell-derived exosomes.

Several mechanisms have been proposed to explain how cells sort small RNAs into exosomes. For example, Villarroya-Beltri *et al.* (2013) proposed that sumoylated hnRNPA2B1 shuttles miRNA with particular sequence motifs into exosomes. Koppers-Lalic *et al.* (2014) reported that 3′ uridylated miRNA isoforms are preferentially loaded into exosomes compared with 3′ adenylated ones. However, neither of these mechanisms accounts for the export of the 14q32 cluster or our differential expression results more generally. While each of these mechanisms may contribute to directing miRNA export, there are clearly other mechanisms accounting for miRNA sorting into exosomes that remain unexplained. Squadrito *et al.* (Squadrito *et al.* 2014) found that both an miRNA’s expression level and that of its targets influences its relative enrichment in exosomes compared with cells, but we lacked the mRNA measurements to evaluate this possibility.

We speculate that the widespread differential expression of miRNA and piRNA, including the export of the 14q32 cluster, is likely driven through sequence recognition by RNA-binding protein(s) with uncharacterized binding affinities. This hypothesis, similar to what has been previously proposed (Villarroya-Beltri *et al.* 2013), could plausibly explain the massive export of 14q32 cluster miRNAs, since they share a consensus sequence untargeted by known RNA-binding proteins. Previous studies have suggested that the miRNA exported in exosomes can silence mRNA in target cells (Hergenreider *et al.* 2012; Montecalvo *et al.* 2012; Umezu *et al.* 2013; van Balkom *et al.* 2013). Given recent evidence that piRNAs, like miRNAs, can silence target mRNAs (Gou *et al.* 2014; Gebert *et al.* 2015), it is possible that both the miRNA and piRNA exported in exosomes serve a regulatory role in recipient cells. Both repression (Sarver *et al.* 2013; Kameswaran *et al.* 2014) and overexpression (Manodoro *et al.* 2014) of the miRNAs in the 14q32 cluster has been associated with disease in various cell types. It is unclear whether the cluster miRNAs have a regulatory role in the target cells or are exported to prevent their activity in the parent cells.

In addition to assessing global differences between cells and exosomes, we sought to quantify the variability seen between individuals, as this has not previously been measured in a sample of this size. We observed that miRNA and piRNA levels varied more between exosome samples than between cell samples. This implies that RNA export in exosomes is less tightly controlled than RNA regulation within cells, which has important implications for the use of exosomes in diagnostics. To investigate one possible source of variability in miRNA and piRNA expression between individuals, we performed the first eQTL analysis of exosomes and their parent cells. We identified the products of hsa-miR-151a and hsa-miR-335 as miRNA eQTLs that appear to be shared between cells and exosomes. It is unsurprising that we only identified a few miRNA eQTLs, since previous studies have suggested that they are rare relative to mRNA eQTLs (Borel *et al.* 2011; Lappalainen *et al.* 2013; Huan *et al.* 2015) and the small sample size limited our statistical power. While it appears that genetic variation can only explain a small portion of the variability in exosomal miRNA between individuals, we found evidence that genetics may play a role in exosome miRNA regulation. We expect that future genetic studies of exosome RNA content will enable the discovery of more miRNA eQTLs, improving upon our identification of genetic differences in intercellular communication and their potential consequences on human phenotypes.

As exosomes are increasingly explored as diagnostic tools and tied to different aspects of human health, it becomes important for us to understand the different levels of regulation they undergo and how their contents may vary from person to person. Our findings improve our understanding of the factors influencing miRNA export and underscore the importance of considering individual differences when studying exosomes.

## Supplementary Material

Supplemental material is available online at www.g3journal.org/lookup/suppl/doi:10.1534/g3.116.036137/-/DC1.
