# Supplementary figures and images for: Small RNA Sequencing in Cells and Exosomes Identifies eQTLs and 14q32 as a Region of Active Export
Source: G3 (Bethesda). 2016 Oct 31;7(1):31–9. doi: 10.1534/g3.116.036137 (PMC5217120; doi:10.1534/g3.116.036137)

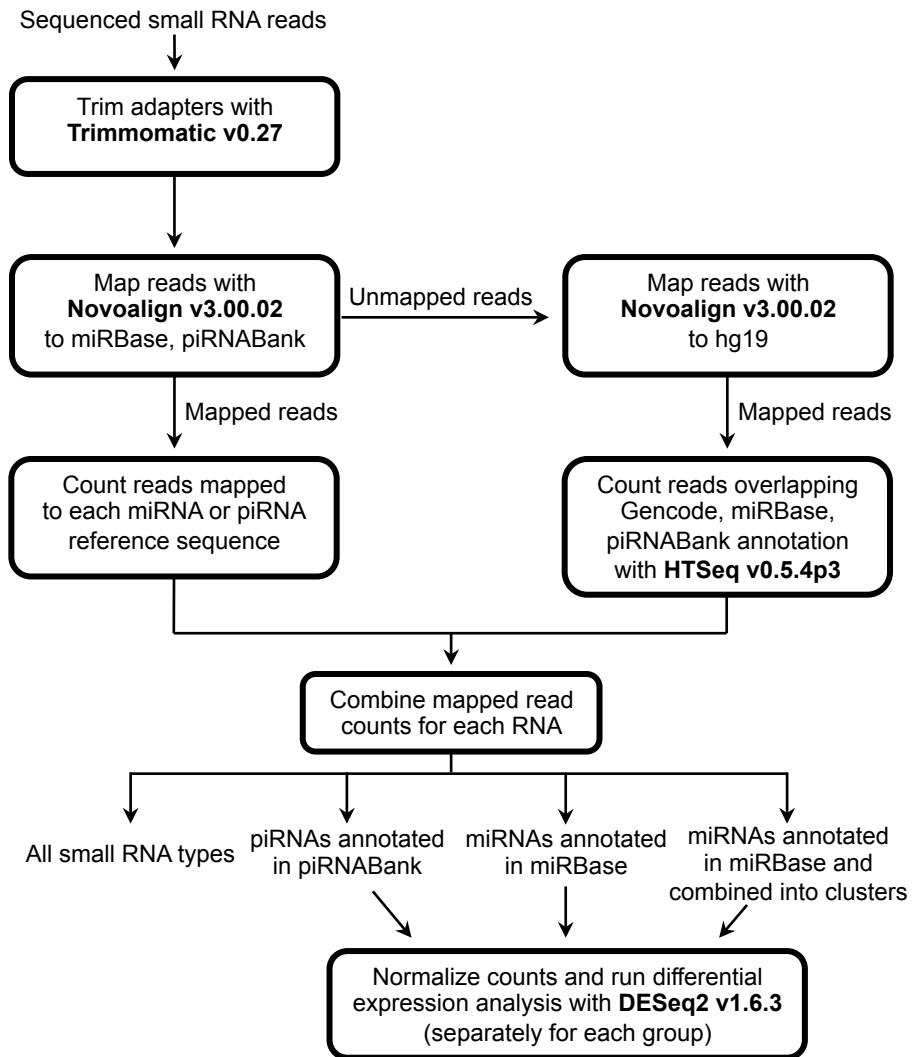

**Figure S1. Flowchart of transcript quantification steps.**

Supplement: Supplementary file 1 [file 31FigureS1.pdf]
