# Supplementary material for: Small RNA Sequencing in Cells and Exosomes Identifies eQTLs and 14q32 as a Region of Active Export
Source: G3 (Bethesda). 2016 Oct 31;7(1):31–9. doi: 10.1534/g3.116.036137 (PMC5217120; doi:10.1534/g3.116.036137)
Supplement: Supplementary file 2 [file 31FigureS2.pdf]

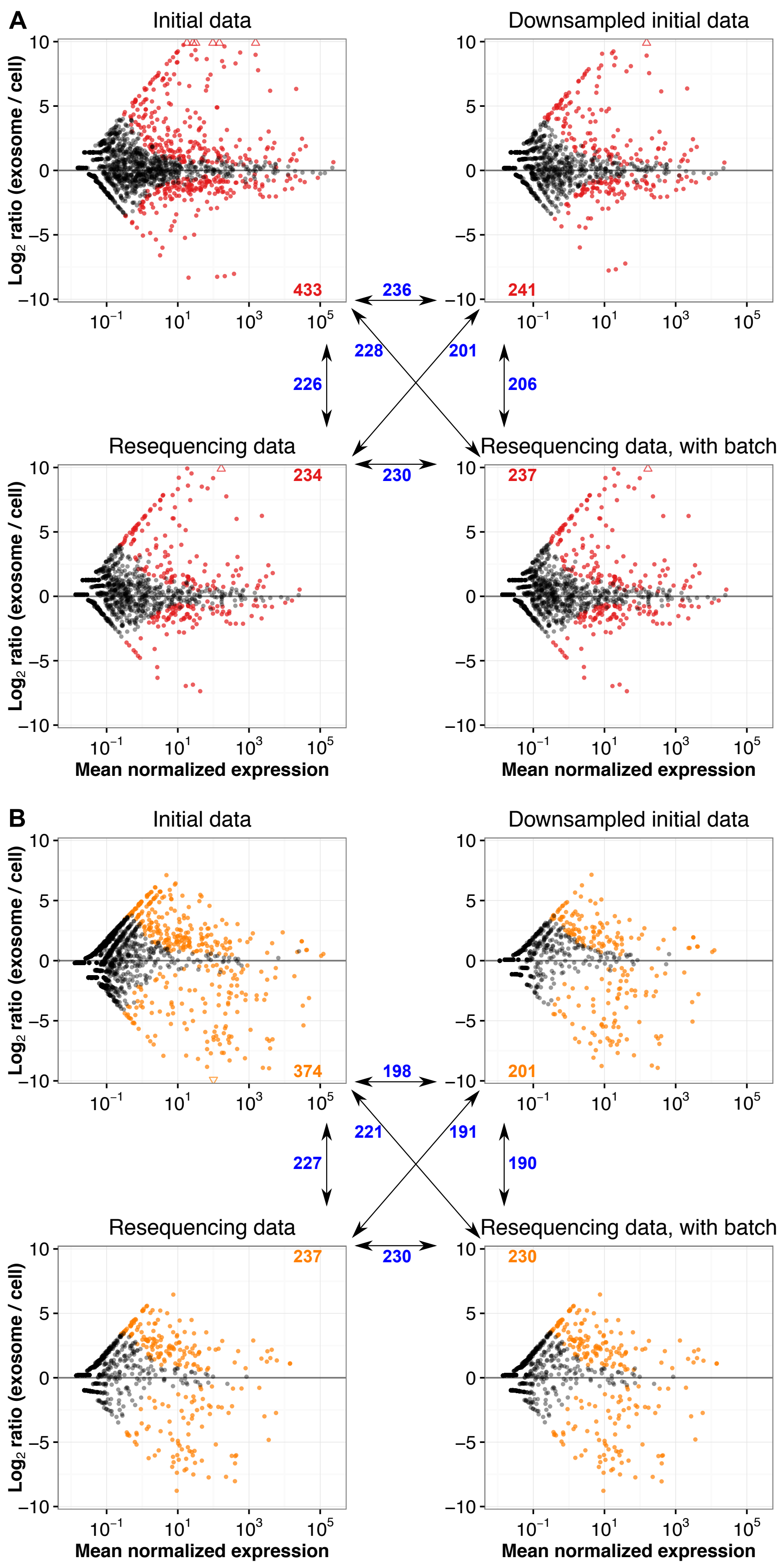

Figure S2. miRNA and piRNA differential expression results replicate in resequencing data. MA plots of initial data compared to the same data downsampled to 10% and to resequencing data with ~10% of the initial sequencing depth (with and without explicitly correcting for batch) for miRNA (A) and piRNA (B). Differentially expressed transcripts are colored and the number of differentially expressed transcripts appears in red or orange in the corner of the plot. The numbers in blue indicate the number of differentially expressed transcripts shared between each pair of analyses. The downsampled data, which roughly match the depth of the resequencing data, give us a sense of (1) the number of differentially expressed transcripts and (2) the degree of overlap with the initial data that we expect from the resequencing data. For both miRNA and piRNA, the pattern of differential expression of the resequencing data matches that of the initial data. Importantly, there is a large overlap between the transcripts called differentially expressed in the initial and the resequencing data. Therefore, the resequencing data confirms that the differential expression seen in the initial data is driven by true differences between cells and exosomes rather than by lane effects.
