# Supplementary material for: Small RNA Sequencing in Cells and Exosomes Identifies eQTLs and 14q32 as a Region of Active Export
Source: G3 (Bethesda). 2016 Oct 31;7(1):31–9. doi: 10.1534/g3.116.036137 (PMC5217120; doi:10.1534/g3.116.036137)
Supplement: Supplementary file 3 [file 31FigureS3.pdf]

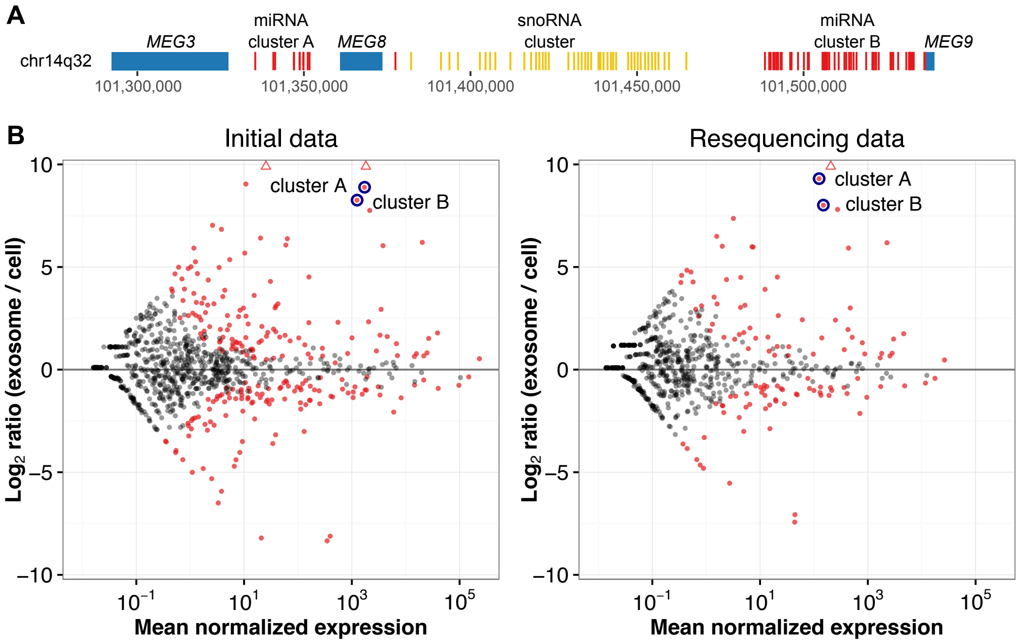

Figure S3. 14q32 miRNA clusters are exosome specific. (A) Gene model of the 14q32 locus showing two miRNA clusters flanked by lincRNAs *MEG3*, *MEG9* and separated by lincRNA *MEG8* and a snoRNA cluster. (B) MA plots of cluster differential expression results for the initial data (left) as well as the resequencing data (right). Each point is a miRNA cluster and the two miRNA clusters on 14q32 are circled in blue. Clusters depicted in red are significantly differentially expressed. Note that some clusters comprise a single miRNA (see Materials and Methods). In both the initial and the resequencing data, the 14q32 miRNA clusters are much more highly expressed in exosomes than cells. In the main text of the paper, we focus on the larger of the two miRNA clusters on 14q32 (cluster B).
