# Supplementary material for: Small RNA Sequencing in Cells and Exosomes Identifies eQTLs and 14q32 as a Region of Active Export
Source: G3 (Bethesda). 2016 Oct 31;7(1):31–9. doi: 10.1534/g3.116.036137 (PMC5217120; doi:10.1534/g3.116.036137)
Supplement: Supplementary file 4 [file 31FigureS4.pdf]

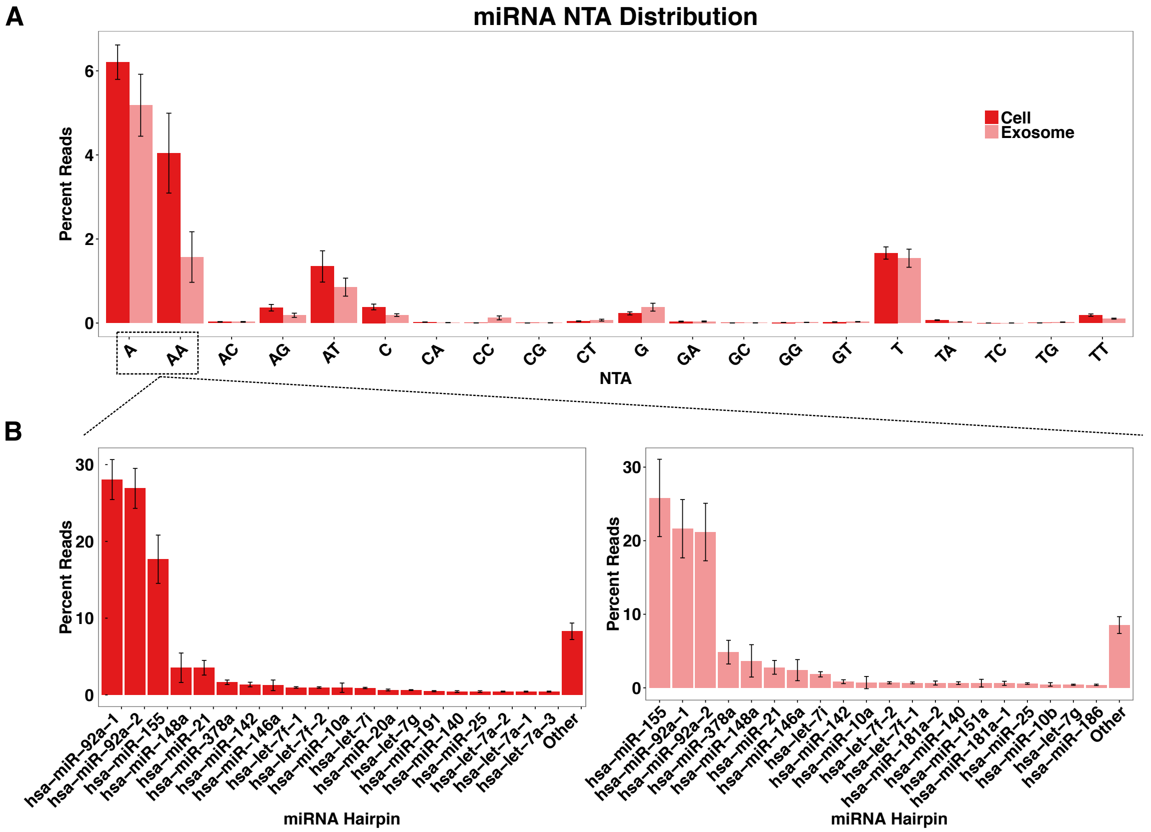

Figure S4. miRNA 3'-NTA distributions are largely driven by extensive modification of a small subset of miRNAs. (A) Distribution of 3'-NTAs as a percentage of all reads mapped to miRBase. On average, ~80% of all reads match the reference exactly. (B) The miRNA hairpin composition, in percentages, of all "-A" and "-AA" NTA reads. In all plots, bars show the mean of the 17 individuals and error bars indicate the standard deviation. Though some 3'-NTAs appear differentially represented in exosomes compared to cells (e.g. "-AA"), these effects are mainly driven by the extensive modification of a small number of miRNAs (for instance, those originating from hsa-miR-92a and hsa-miR-155) rather than widespread modification of many different miRNAs.
