# Supplementary material for: Small RNA Sequencing in Cells and Exosomes Identifies eQTLs and 14q32 as a Region of Active Export
Source: G3 (Bethesda). 2016 Oct 31;7(1):31–9. doi: 10.1534/g3.116.036137 (PMC5217120; doi:10.1534/g3.116.036137)
Supplement: Supplementary file 5 [file 31FigureS5.pdf]

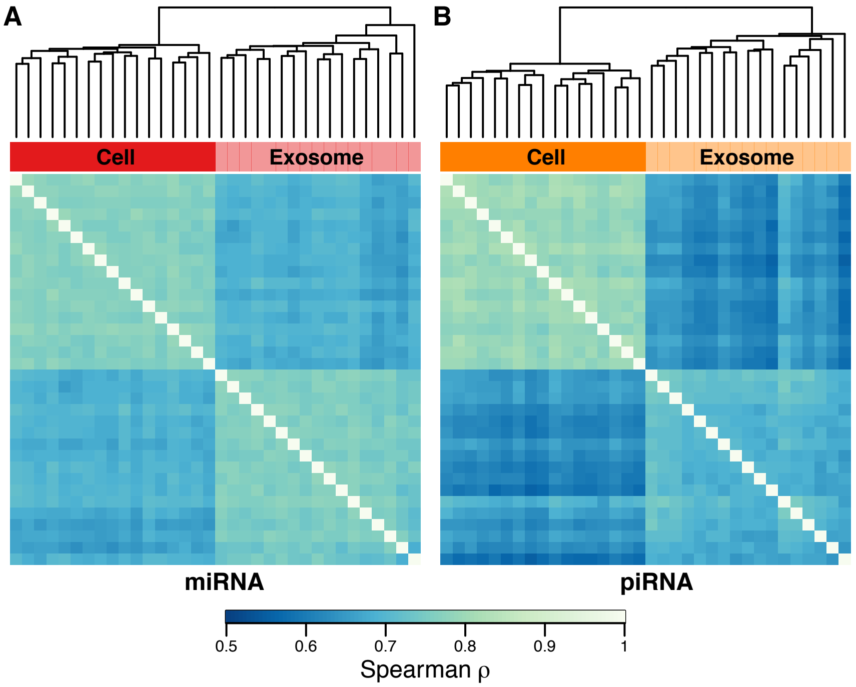

Figure S5. The clustering of cells and exosomes by their expression profiles replicates in the resequencing data. Hierarchical clustering of resequenced samples by their Spearman correlation coefficients for both miRNA (A) and piRNA (B). As in the initial sequencing data, the average correlations were higher between cell samples than between exosome samples (two-sided Wilcoxon rank sum test, miRNA 0.77 vs. 0.75, $p = 3.3 \times 10^{-11}$; piRNA 0.79 vs. 0.70, $p < 1 \times 10^{-15}$).
