# Supplementary material for: Small RNA Sequencing in Cells and Exosomes Identifies eQTLs and 14q32 as a Region of Active Export
Source: G3 (Bethesda). 2016 Oct 31;7(1):31–9. doi: 10.1534/g3.116.036137 (PMC5217120; doi:10.1534/g3.116.036137)
Supplement: Supplementary file 6 [file 31FigureS6.pdf]

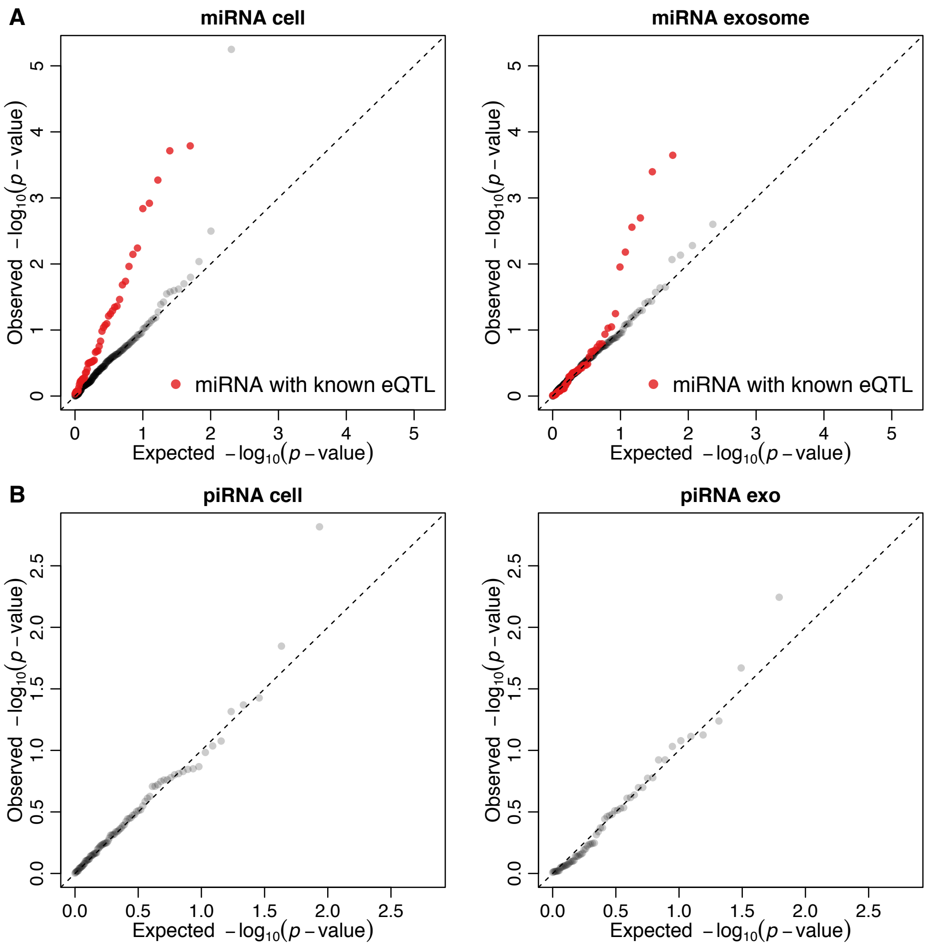

Figure S6. Small RNA eQTL tests detect known miRNA eQTLs in cells but show no signal for piRNA eQTLs. (A,B) QQ-plots compare observed -$\log_{10}$ *p*-values of the eQTL tests with the expected values from a uniform distribution. (A) miRNA eQTL QQ-plots show an excess of small *p*-values in cells (left) with a weaker signal in exosomes (right). The subset of points corresponding to miRNAs with known eQTLs at a 5% FDR from two large cohort studies, one from 373 LCLs (Lappalainen *et al.* 2013) and another from 5239 whole blood samples (Huan *et al.* 2015), are colored in red. The miRNAs with known eQTLs are significantly enriched toward the lower end of the *p*-value distribution for cells (two-sided Wilcoxon rank-sum; $p = 9.5 \times 10^{-5}$), which indicates that we are replicating known effects. The enrichment is not statistically significant in exosomes ($p = 0.79$). (B) QQ-plots for piRNA eQTL tests show no strong enrichment of small *p*-values in cells (left) nor exosomes (right).
