# Supplementary material for: Small RNA Sequencing in Cells and Exosomes Identifies eQTLs and 14q32 as a Region of Active Export
Source: G3 (Bethesda). 2016 Oct 31;7(1):31–9. doi: 10.1534/g3.116.036137 (PMC5217120; doi:10.1534/g3.116.036137)
Supplement: Supplementary file 7 [file 31FigureS7.pdf]

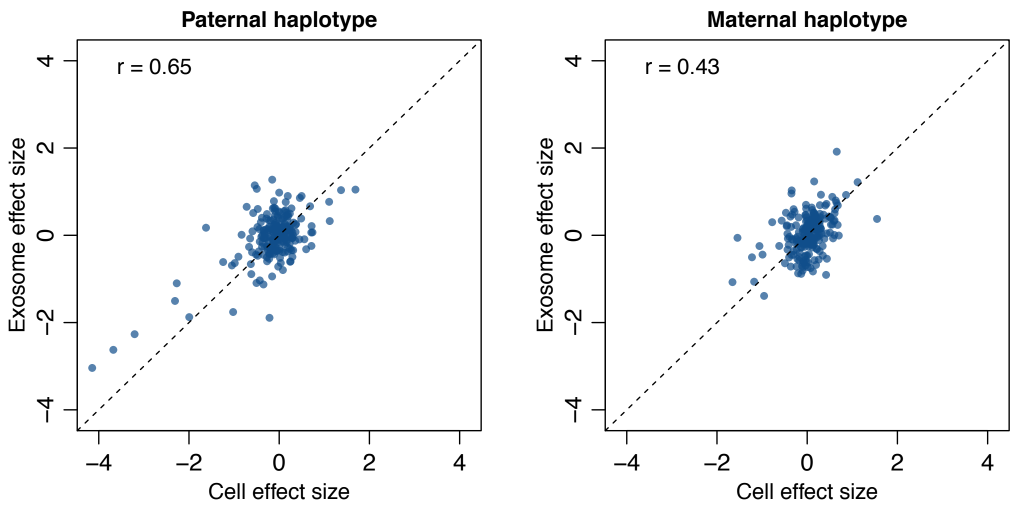

Figure S7. Cell and exosome miRNA eQTL effect sizes are correlated. For miRNAs that were expressed in both cells and exosomes, we compared the miRNA eQTL effects sizes for the paternal (left) and maternal (right) haplotypes using Pearson's correlation. Correlations are indicated directly on the plots and were significant for both parental haplotypes (paternal, $p < 1 \times 10^{-15}$; maternal $p = 3.06 \times 10^{-11}$).
