# Supplementary material for: Small RNA Sequencing in Cells and Exosomes Identifies eQTLs and 14q32 as a Region of Active Export
Source: G3 (Bethesda). 2016 Oct 31;7(1):31–9. doi: 10.1534/g3.116.036137 (PMC5217120; doi:10.1534/g3.116.036137)
Supplement: Supplementary file 8 [file 31FileS1.pdf]

Table S1. Sequenced and mapped read counts per sample. For each sample, the number of reads sequenced, remaining after adapter trimming, and mapped in each alignment step. For reads mapping to hg19 (second alignment step), we also provide the number of reads that mapped to multiple locations, ambiguously mapped to multiple transcripts, or were mapped to unannotated regions.

Table S2. DESeq2 Differential expression results. (A) DESeq2 results for individual miRNAs annotated in miRBase. (B) DESeq2 results for individual piRNAs. (C) DESeq2 results for miRNA clusters. Each table contains the mean normalized counts across all samples (baseMean), the $\log_2$ ratios (exosome / cell) (log2FoldChange), the standard errors on the $\log_2$ ratios (lfcSE), the Wald statistics (stat), the nominal *p*-values (pvalue), and the Benjamini-Hochberg-adjusted *p*-values (padj). Note that transcripts or clusters that were excluded by independent filtering have "NA" in the padj column (see Love *et al.* 2014 for details). (D) Cluster ownership information. The table includes, for each miRNA, the number of clusters it maps to and the name(s) of the cluster(s). The cluster names contain the strand and genomic location as well as the number of miRNAs within that cluster.

Table S3. eQTL results for miRNAs, piRNAs and their respective haplotypes. (A) miRNA eQTL results. (B) piRNA eQTL results. Each table contains the haplotype's identifier and genomic location, the paternal and maternal haplotypes for each child (e.g. "NA12879p" and "NA12879m"), the RNA's identifier and genomic location, the normalized expression values in cells and exosomes for each child (e.g. "NA12879cell" and "NA12879exosome"), and the statistics from the linear model (see table below). For miRNAs (A), the table also includes in which study, if any, the miRNA was identified as an eQTL.

Statistics from the linear model (provided for both cells and exosomes)

Column name	Description
R2	Fit of the linear model
pval	*p*-value on the model fit
BetaP	Paternal effect size
BetaM	Maternal effect size
padj	Benjamini-Hochberg-adjusted *p*-value
